# Addressing Psychiatric Symptoms in Wilson’s Disease: Translational Overlap with Bipolar Disorder and Emerging Therapeutic Strategies

**DOI:** 10.3390/jcm14165866

**Published:** 2025-08-19

**Authors:** Mauro Giovanni Carta, Paula C. Zimbrean, Massimo Claudio Fantini, Diego Primavera

**Affiliations:** 1 Department of Medical Sciences and Public Health, University of Cagliari, 09124 Cagliari, Italy; maurogcarta@gmail.com (M.G.C.); massimoc.fantini@unica.it (M.C.F.); 2 Departments of Psychiatry and Surgery (Transplant), Yale School of Medicine, New Haven, CT 06510, USA; paula.zimbrean@yale.edu

**Keywords:** Wilson’s disease, psychiatric manifestations, bipolar disorder, copper neurotoxicity, oxidative stress, circadian rhythm

## Abstract

**Background:** Wilson’s disease (WD) is a rare autosomal recessive disorder characterized by abnormal copper metabolism and accumulation in the liver and brain. While hepatic and neurological manifestations are well-recognized, psychiatric symptoms remain underdiagnosed and frequently precede other clinical signs, leading to delayed diagnosis and poorer outcomes. **Objective:** This opinion paper aims to explore the emerging understanding of psychiatric features in WD, particularly mood disturbances and their overlap with bipolar spectrum disorders, through a translational lens. **Opinion:** Psychiatric manifestations—including irritability, aggression, disinhibition, and mood instability—are observed in up to 100% of symptomatic WD patients. Accumulated copper induces oxidative stress and astrocyte dysfunction, which may disrupt neural circuits involved in emotion regulation. There is increasing evidence of shared pathophysiological mechanisms between WD and bipolar disorder, including redox imbalance and circadian rhythm dysregulation. **Future Directions:** The timely recognition of psychiatric symptoms is essential. Future research should investigate biomarkers of early psychiatric involvement, evaluate psychotropic medication safety in WD, and implement psychoeducational strategies to improve adherence and quality of life. A translational approach can foster individualized interventions and provide insights into broader mood disorders.

## 1. Introduction

Wilson’s disease (WD) is an autosomal recessive hereditary multisystem disorder characterized by abnormal copper metabolism. The disease leads to progressive copper accumulation in various organs, with the liver being the initial site of toxicity, followed by involvement of the central nervous system. This progression results in a wide spectrum of hepatic, neurological, and psychiatric manifestations [1].

The estimated prevalence in the general population ranges from 1 in 30,000 to 1 in 100,000 individuals, with a carrier frequency of approximately 1 in 90. A positive family history is found in 25% to 50% of diagnosed cases, consistent with the autosomal recessive pattern of inheritance [2].

As with many hereditary disorders, the incidence of WD is significantly higher in geographically or genetically isolated populations with high rates of consanguinity. A well-documented example is Sardinia, an Italian island, where the observed incidence reaches 1 in 7000 live births [3].

Furthermore, some genetic studies suggest that the actual genetic prevalence of WD may be three to four times higher than the clinically diagnosed prevalence, indicating that the penetrance of ATP7B mutations may not be complete, contrary to earlier assumptions [2].

## 2. Pathophysiological Basis of Psychiatric Symptoms in Wilson’s Disease

### 2.1. Pathogenesis and Mechanisms of Copper Accumulation

The genetic mutation responsible for Wilson’s disease is located on the short arm of chromosome 13 and affects the *ATP7B* gene. This gene encodes a copper-transporting P-type ATPase, which plays a key role in hepatocellular copper regulation. Under physiological conditions, ATP7B facilitates the excretion of excess copper into bile and the incorporation of copper into apoceruloplasmin to form functional holoceruloplasmin.

In WD, mutations in ATP7B impair both the biliary excretion of copper and the proper loading of copper onto ceruloplasmin. This results in the formation of apoceruloplasmin, a non-functional form of the protein, and the accumulation of non-ceruloplasmin-bound copper in the bloodstream, which is highly toxic.

This free copper progressively accumulates in target organs—most notably the liver, brain, cornea, and kidneys—leading to widespread cellular damage. Due to its redox-active properties, intracellular copper contributes to oxidative stress, which damages lipids, proteins, and nucleic acids. This oxidative injury underlies the pathophysiological mechanisms responsible for the hepatic dysfunction, neurodegeneration, and psychiatric symptoms commonly seen in WD [4,5].

### 2.2. Neuronal Oxidative Stress (OxS)

In Wilson’s disease, the accumulation of free copper leads to significant oxidative stress (OxS) in neural tissues, a central mechanism contributing to both neurological and psychiatric symptoms. Oxidative stress disrupts neuronal homeostasis by impairing signal transduction pathways, reducing synaptic plasticity, and compromising neuronal survival. The process is mainly mediated by lipid peroxidation of neuronal membranes, oxidation of structural and functional proteins, and DNA damage [6].

Excess non-ceruloplasmin-bound copper in WD acts as a redox-active ion, generating reactive oxygen species (ROS) that exacerbate neuronal injury. This condition has been shown to activate pro-inflammatory cytokines and contribute to glutamate excitotoxicity, further damaging neurons through sustained overstimulation of NMDA receptors [7,8]. Such biochemical cascades are particularly detrimental in brain regions critical for emotional regulation and behavior.

Astrocytes, key regulators of the blood–brain barrier and neuronal environment, initially buffer excess extracellular copper by increasing intracellular sequestration. This response leads to astrogliosis, characterized by astrocyte proliferation, cellular swelling, and upregulation of metallothioneins—copper-binding proteins that aim to limit free copper toxicity [9,10]. However, prolonged copper overload overwhelms these compensatory mechanisms, altering astrocyte function and the neuronal microenvironment. Disruption of astrocytic activity results in impaired neuronal support, metabolic dysfunction, and loss of glutamate reuptake capacity, contributing to excitotoxic damage [11].

These neurotoxic processes affect specific brain circuits involved in mood regulation, motor behavior, and reward processing, such as the basal ganglia, thalamus, and limbic structures—regions also implicated in the pathophysiology of bipolar disorder (BD) [12,13]. Several studies on BD have demonstrated altered oxidative stress parameters, including changes in levels of superoxide dismutase (SOD), catalase (CAT), and thiobarbituric acid reactive substances (TBARSs), suggesting that redox imbalance may be a shared pathophysiological substrate between BD and WD [14].

Copper deposition in WD is particularly evident in the basal ganglia, but other regions such as the thalamus, cerebellum, and cortical areas may also be involved. Clinically, this correlates with the frequent occurrence of neurological symptoms, including tremor, dystonia, dysarthria, dysphagia, sialorrhea, cognitive impairment, and diverse psychiatric manifestations [15].

### 2.3. Psychiatric Manifestations

The mechanisms underlying psychiatric symptoms in Wilson’s disease remain incompletely understood. Evidence suggests that copper neurotoxicity within the central nervous system plays a central role, as supported by case reports describing depressive and other psychiatric symptoms coinciding with cerebral copper accumulation [16].

Recent studies have proposed that elevated copper levels, similar to other heavy metals and trace elements such as zinc, cadmium, and thallium, may contribute to the neurodegenerative processes underlying bipolar disorder. This supports the hypothesis that metal-induced oxidative stress and neuroinflammation are critical components in the development of mood dysregulation [17].

In addition to copper-induced neurotoxicity, hepatic encephalopathy (HE) may also play a role in WD-associated psychiatric disturbances. Liver cirrhosis leads to porto-systemic shunting, which permits the entry of gut-derived neurotoxins (e.g., ammonia, mercaptans, and short-chain fatty acids) into the brain, further exacerbating neuropsychiatric symptoms through glial activation and altered neurotransmission [18].

### 2.4. Wilson’s Disease and Psychiatric Disorders

Psychiatric symptoms in Wilson’s disease may emerge before, during, or after the formal diagnosis, and frequently precede neurological signs, often leading to delayed recognition of the disease. Retrospective analyses report that up to 20% of WD patients had previously consulted a psychiatrist, and 30–40% exhibit psychiatric symptoms at the time of diagnosis [18,19]. Overall, psychiatric disturbances are reported in 30% to 100% of symptomatic patients, highlighting their variable and often subtle presentation [20].

In his seminal 1912 publication, S.A.K. Wilson described psychiatric involvement in 66.6% of patients, including schizophrenia-like psychosis in 16.6% of cases [21]. Contemporary clinical profiles, however, more frequently involve affective symptoms, such as irritability, depression, mood lability, and suicidal ideation. Personality changes, sexual disinhibition, and impulsivity are also prominent [22].

Patients with WD may also experience cognitive dysfunction, psychosis, anxiety disorders, and in some cases, mood disorders within the bipolar spectrum, including mania, hypomania, agitation, aggression, and behavioral disinhibition [23]. These features may mirror primary psychiatric disorders but are likely secondary to copper dysregulation and oxidative stress. A growing body of literature suggests a clinical and biological overlap between BD and WD, with shared markers such as altered redox status and circadian rhythm disruption [24,25,26].

Sleep disorders are another common and underappreciated aspect of WD. Reported in 40–80% of patients, they span across hepatic and neurological phenotypes and include insomnia, hypersomnia, circadian rhythm disturbances, parasomnias, and sleep-related movement disorders [27]. Although the precise impact of these sleep alterations on psychiatric outcomes in WD remains unclear, their presence likely exacerbates mood instability and cognitive impairments.

## 3. Expert Opinion

Wilson’s disease (WD) is a complex multisystem disorder that poses significant diagnostic and therapeutic challenges due to its protean manifestations across hepatic, neurological, and psychiatric domains. Given its variable phenotype, WD can mimic a wide range of internal medicine and psychiatric conditions, often leading to misdiagnosis or delayed recognition. This raises the ongoing debate as to whether WD should be conceptualized as a single disease entity or rather as a spectrum of related disorders linked by a common genetic and pathophysiological substrate [28].

Psychiatric symptoms may be the initial presentation of WD, especially in younger patients without overt hepatic or neurological signs. In some cases, these symptoms precede the diagnosis by months or even years. A purely psychiatric onset is rare but particularly problematic, as it may delay appropriate investigations. Early psychiatric features can include declining academic performance, personality changes, irritability, or depression, all of which are often mistakenly attributed to primary psychiatric disorders [29].

Historically, psychiatric manifestations in WD were predominantly characterized as schizophrenia-like symptoms, a description that likely reflected both the diagnostic limitations of the early 20th century and the advanced disease stages at which patients were typically diagnosed [21]. Two key factors explain the shift in clinical presentation observed today: (1) the refinement of psychiatric diagnostic criteria, particularly following the publication of the DSM-III, which narrowed the scope of schizophrenia diagnosis, and (2) the earlier detection of WD, which now captures patients before extensive neurodegeneration occurs. Thus, previous observations likely reflected cases in which severe copper-induced oxidative damage led to necrotic brain lesions and more florid psychiatric syndromes, now seldom seen with timely intervention.

Currently, mood disorders—particularly those within the bipolar spectrum—represent the most common psychiatric manifestations of WD [30]. Symptoms such as mood lability, impulsivity, irritability, aggression, and hyperactivity often occur in conjunction with neurological findings and are supported by neuroimaging abnormalities, particularly in the basal ganglia and thalamus. These findings suggest a neurobiological basis for emotional dysregulation in WD.

Prompt diagnosis and consistent treatment adherence are critical to ensuring favorable long-term outcomes. Patients with early-stage WD who receive timely therapy often maintain normal life expectancy and quality of life (QoL) [31]. However, when psychiatric symptoms are prominent, the average diagnostic delay exceeds two years, with some cases reporting delays of up to 12 years [32]. Delayed diagnosis increases the risk of irreversible organ damage and poor psychosocial functioning.

Quality of life in WD appears closely related to psychiatric status, particularly the presence of depressive symptoms. Interestingly, some studies indicate that QoL is higher among women and among patients with hepato-neurological phenotypes, although the mechanisms remain unclear [20].

Psychiatric manifestations in WD also have a profound impact on families and caregivers, as they complicate disease management and may jeopardize treatment adherence. Patients with psychiatric symptoms often exhibit poor compliance with both anti-copper therapy and psychotropic medications, either due to cognitive impairment, lack of insight, or side-effects. This underlines the importance of a multidisciplinary approach, including psychoeducation programs for both patients and families. Psychoeducation serves as a critical tool, not only to enhance adherence and coping strategies but also to improve the understanding of the disease trajectory, lifestyle modifications (e.g., diet, physical activity), and symptom monitoring [33].

Upon diagnosis, patients must initiate lifelong copper chelation therapy, accompanied by a low-copper diet. Zinc monotherapy is often preferred due to its minimal neurological side-effects, particularly in asymptomatic or pre-symptomatic individuals [22,31]. During the initial phase of treatment, neuropsychiatric symptoms may worsen transiently as tissue-bound copper is mobilized into circulation.

The pharmacological management of psychiatric symptoms in Wilson’s disease (WD) is primarily based on case reports, case series, and expert consensus, as no randomized controlled trials (RCTs) have been conducted to date in this specific population [20,22,34]. The EASL Clinical Practice Guidelines (2012) [22] provide general treatment recommendations for WD but offer limited guidance on psychiatric care. A critical evaluation of the risk–benefit profile of individual psychotropic agents—particularly in relation to hepatic and neurological vulnerability—is essential in clinical decision making [34].

Second-generation antipsychotics such as quetiapine or olanzapine are generally preferred due to their lower extrapyramidal side-effect profile, although caution is warranted given the risk of metabolic disturbances. First-generation antipsychotics (e.g., haloperidol) should be avoided when possible, due to their potential to exacerbate neurological symptoms [34,35].

Among mood stabilizers, lithium is considered effective in selected cases but requires close monitoring for renal function, especially in patients with renal tubular dysfunction. Lamotrigine may represent a safer alternative due to its hepatic safety profile and antioxidant potential [35]. Benzodiazepines such as lorazepam are commonly used for acute agitation or anxiety, given their minimal hepatic metabolism, although long-term use should be avoided [20].

Finally, there is increasing interest in repositioning psychotropic agents with antioxidant or circadian-stabilizing properties—such as N-acetylcysteine or agomelatine—based on translational parallels with mood disorders involving redox imbalance and neuroinflammation [35].

Ultimately, adherence to anti-copper therapy, together with careful management of psychiatric symptoms, enables most patients with WD to achieve a good prognosis. Comprehensive diagnostic assessment and the implementation of structured psychoeducational interventions are essential components of effective long-term management. Future research should aim to clarify the underlying neurobiological mechanisms linking copper toxicity, oxidative stress, and psychiatric disorders in WD, and to identify personalized treatment strategies that address both the somatic and mental health needs of these patients [23]. A schematic summary of the proposed pathophysiological pathways linking copper accumulation to neuropsychiatric symptoms is provided in Table 1.

## 4. Future Directions

The neuropsychiatric manifestations of Wilson’s disease (WD) remain an underexplored frontier of clinical research, despite their high prevalence and significant impact on patient prognosis and quality of life. Several important avenues for future investigation can be identified.

First, longitudinal studies are urgently needed to clarify the temporal relationship between copper accumulation, oxidative stress markers, and the onset of specific psychiatric symptoms. Integrating clinical data with advanced neuroimaging and biomarker analysis (e.g., serum-free copper, ceruloplasmin, and redox enzymes) may help delineate early predictors of psychiatric involvement.

Second, given the clinical overlap with bipolar disorder, future research should investigate shared pathophysiological mechanisms, particularly oxidative imbalance, neuroinflammation, and circadian rhythm dysregulation. This could open the door to repurposing psychotropic agents with antioxidant or circadian-stabilizing properties for WD-associated mood disorders. Based on shared pathophysiological mechanisms between Wilson’s disease (WD) and mood disorders—including oxidative stress, neuroinflammation, and astrocytic dysfunction—it is reasonable to hypothesize that psychotropic agents with antioxidant and chronobiotic properties may offer therapeutic benefit. This approach aligns with the increasing interest in a translational framework that links molecular dysfunctions to targeted interventions. For instance, lamotrigine and lithium, both used as mood stabilizers, have shown potential in modulating oxidative and inflammatory pathways, but must be prescribed with caution in WD due to hepatic and renal vulnerabilities [35]. Moreover, N-acetylcysteine (NAC), a glutathione precursor with well-documented antioxidant and glutamatergic modulating properties, has demonstrated efficacy in mood disorders such as bipolar depression and may be particularly promising for WD-related psychiatric symptoms [36]. NAC could help buffer oxidative damage induced by copper accumulation, potentially stabilizing emotional dysregulation and improving cognitive outcomes. Although no clinical studies have tested NAC in WD, its safety profile and neuroprotective mechanisms justify future translational research [36]. Similarly, melatonin agonists such as agomelatine, already explored in the treatment of bipolar disorder, may offer dual benefits via circadian rhythm stabilization and anti-inflammatory effects [37].

Third, interventional studies are needed to evaluate the efficacy and safety of specific psychiatric treatments in WD populations, particularly in relation to hepatic and renal tolerability. Psychotropic medications should be tested not only for their symptom control but also for their impact on treatment adherence and functional outcomes.

Finally, the role of psychoeducation and family-based interventions should be more systematically explored. Tailored psychoeducational programs may help improve insight, reduce stigma, and optimize adherence, especially in patients with psychiatric-onset WD.

A deeper understanding of the neuropsychiatric aspects of WD will not only refine diagnostic and therapeutic strategies but also provide insights into broader questions regarding the neurobiology of mood and behavior.

## Figures and Tables

**Table 1:** Pathophysiological mechanisms potentially underlying psychiatric symptoms in Wilson’s disease (WD).

Mechanism	Pathological Process	Neuropsychiatric Impact	Potential Targets/Implications
Copper accumulation	ATP7B mutation leads to impaired biliary copper excretion and systemic accumulation	Direct neurotoxicity, especially in basal ganglia and limbic system	Chelation therapy (e.g., D-penicillamine, trientine), zinc supplementation
Oxidative stress	Copper catalyzes production of reactive oxygen species (ROS)	Neuronal damage, mood dysregulation, cognitive dysfunction	Antioxidant agents (e.g., N-acetylcysteine, lamotrigine)
Mitochondrial dysfunction	ROS and copper disrupt mitochondrial energy metabolism	Fatigue, emotional instability, impaired synaptic plasticity	Mitochondrial protectants, mood stabilizers
Glutamate excitotoxicity	Impaired astrocytic reuptake and increased extracellular glutamate levels	Anxiety, agitation, psychosis, cognitive deficits	Glutamate modulators (experimental), mood stabilizers
Astrocyte dysfunction	Loss of astrocytic regulation of synaptic homeostasis and neurovascular coupling	Worsening of glutamatergic toxicity and inflammation	Translational marker; indirect target of antioxidant therapies
Neuroinflammation	Activation of microglia and inflammatory cytokine release	Depression-like behavior, neurodegeneration	Anti-inflammatory strategies (investigational)
Basal ganglia degeneration	Copper-induced damage to striatum and globus pallidus	Extrapyramidal symptoms, affective lability, executive dysfunction	Basis for avoiding first-generation antipsychotics
Circadian rhythm dysregulation	Disruption of suprachiasmatic nucleus and melatonin signaling	Sleep disturbances, mood cycling, emotional instability	Chronobiotics (e.g., agomelatine, melatonin), lifestyle interventions

## References

[1] Bearn A.G. (1959). A genetical analysis of thirty families with Wilson’s disease (hepatolenticular degeneration). Ann. Hum. Genet..

[2] Sandahl T.D., Laursen T.L., Munk D.E., Vilstrup H., Weiss K.H., Ott P. (2020). The Prevalence of Wilson’s Disease: An Update. Hepatology.

[3] Loudianos G., Dessi V., Lovicu M., Angius A., Figus A., Lilliu F., De Virgiliis S., Nurchi A.M., Ala A., Walker A.P. (2007). Wilson’s disease. Lancet.

[4] de Bie P., Muller P., Wijmenga C., Klomp L.W.J. (2007). Molecular pathogenesis of Wilson and Menkes disease: Correlation of mutations with molecular defects and disease phenotypes. J. Med. Genet..

[5] Lalioti V., Sandoval I., Cassio D., Duclos-Vallée J.-C. (2010). Molecular pathology of Wilson’s disease: A brief. J. Hepatol..

[6] Sathyanarayana Rao T.S., Zecca L., Jagannatha Rao K. (2007). Tracemetals, neuromelanin and neurodegeneration: An interesting area for research. Indian J. Psychiatry.

[7] Nagasaka H., Takayanagi M., Tsukahara H. (2009). Children’s toxicology from bench to bed—Liver Injury (3): Oxidative stress and anti-oxidant systems in liver of patients with wilson disease. J. Toxicol. Sci..

[8] Kalita J., Kumar V., Misra U. (2015). A Study on Apoptosis and Anti-apoptotic Status in Wilson Disease. Mol. Neurobiol..

[9] Scheiber I.F., Dringen R. (2011). Copper-treatment increases the cellular GSH content and accelerates GSH export from cultured rat astrocytes. Neurosci. Lett..

[10] Bertrand E., Lewandowska E., Szpak G.M., Hoogenraad T., Blaauwgers H.G., Członkowska A., Dymecki J. (2001). Neuropathological analysis of pathological forms of astroglia in Wilson’s disease. Folia Neuropathol..

[11] Pal A., Prasad R. (2014). Recent Discoveries on the Functions of Astrocytes in the Copper Homeostasis of the Brain: A Brief Update. Neurotox. Res..

[12] Machado-Vieira R., Andreazza A.C., Viale C.I., Zanatto V., Cereser V., Vargas R.d.S., Kapczinski F., Portela L.V., Souza D.O., Salvador M. (2007). Oxidative stress parameters in unmedicated and treated bipolar subjects during initial manic episode: A possible role for lithium antioxidant effects. Neurosci. Lett..

[13] Zarate C.A., Singh J., Manji H.K. (2006). Cellular Plasticity Cascades: Targets for the Development of Novel Therapeutics for Bipolar Disorder. Biol. Psychiatry.

[14] Andreazza A.C., Cassini C., Rosa A.R., Leite M.C., de Almeida L.M., Nardin P., Cunha A.B., Ceresér K.M., Santin A., Gottfried C. (2007). Serum S100B and antioxidant enzymes in bipolar patients. J. Psychiatr. Res..

[15] Penning L.C., Berenguer M., Czlonkowska A., Double K.L., Dusek P., Espinós C., Lutsenko S., Medici V., Papenthin W., Stremmel W. (2023). A Century of Progress on Wilson Disease and the Enduring Challenges of Genetics, Diagnosis, and Treatment. Biomedicines.

[16] Jukić I., Titlić M., Tonkić A., Dodig G., Rogosić V. (2006). Psychosis and Wilson’s disease: A case report. Psychiatr. Danub..

[17] González-Estecha M., Trasobares E.M., Tajima K., Cano S., Fernández C., López J.L., Unzeta B., Arroyo M., Fuentenebro F. (2011). Trace elements in bipolar disorder. J. Trace Elements Med. Biol..

[18] Scheiber I.F., Brůha R., Dušek P. (2017). Pathogenesis of Wilson disease. Handb. Clin. Neurol..

[19] Akil M., Brewer G.J. (1995). Psychiatric and behavioral abnormalities in Wilson’s disease. Adv. Neurol..

[20] Zimbrean P.C., Schilsky M.L. (2014). Psychiatric aspects of Wilson disease: A review. Gen. Hosp. Psychiatry.

[21] Wilson S.A.K. (1912). Progressive lenticular degeneration: A familial nervous disease associated with cirrhosis of the liver. Brain.

[22] European Association for the Study of the Liver (2012). EASL Clinical Practice Guidelines: Wilson’s disease. J. Hepatol..

[23] Mura G., Zimbrean P.C., Demelia L., Carta M.G. (2017). Psychiatric comorbidity in Wilson’s disease. Int. Rev. Psychiatry.

[24] Carta M.G., Sorbello O., Moro M.F., Bhat K.M., Demelia E., Serra A., Mura G., Sancassiani F., Piga M., Demelia L. (2012). Bipolar disorders and Wilson’s disease. BMC Psychiatry.

[25] Carta M.G., Kalcev G., Fornaro M., Pinna S., Gonzalez C.I.A., Nardi A.E., Primavera D. (2023). Does Screening for Bipolar Disorders Identify a “Dysregulation of Mood, Energy, and Social Rhythms Syndrome” (DYMERS)? A Heuristic Working Hypothesis. J. Clin. Med..

[26] Carta M.G., Kalcev G., Scano A., Pinna S., Gonzalez C.I.A., Nardi A.E., Orrù G., Primavera D. (2023). Screening, Genetic Variants, and Bipolar Disorders: Can Useful Hypotheses Arise from the Sum of Partial Failures?. Clin. Pract..

[27] Cochen De Cock V., Woimant F., Poujois A. (2019). Sleep Disorders in Wilson’s Disease. Curr. Neurol. Neurosci. Rep..

[28] Shanmugiah A., Sinha S., Taly A.B., Prashanth L.K., Tomar M., Arunodaya G.R., Janardhana Y.C., Khanna S. (2008). Psychiatric manifestations in Wilson’s disease: A cross-sectional analysis. J. Neuropsychiatry Clin. Neurosci..

[29] Cleymaet S., Nagayoshi K., Gettings E., Faden J. (2019). A review and update on the diagnosis and treatment of neuropsychiatric Wilson disease. Expert Rev. Neurother..

[30] Poujois A., Woimant F., Samson S., Chaine P., Girardot-Tinant N., Tuppin P. (2018). Characteristics and prevalence of Wilson’s disease: A 2013 observational population-based study in France. Clin. Res. Hepatol. Gastroenterol..

[31] Roberts E.A., Schilsky M.L. (2008). Diagnosis and treatment of Wilson disease: An update. Hepatology.

[32] Chevalier K., Rahli D., de Veyrac L., Guillaume J., Obadia M.A., Poujois A. (2023). Quality of life and depression in Wilson’s disease: A large prospective cross-sectional study. Orphanet J. Rare Dis..

[33] Lacerda G., Krummel T., Sabourdy C., Ryvlin P., Hirsch E. (2006). Optimizing therapy of seizures in patients with renal or hepatic dysfunction. Neurology.

[34] Litwin T., Dusek P., Szafrański T., Dzieżyc K., Członkowska A., Rybakowski J.K. (2018). Psychiatric manifestations in Wilson’s disease: Possibilities and difficulties for treatment. Ther. Adv. Psychopharmacol..

[35] Gromadzka G., Antos A., Sorysz Z., Litwin T. (2024). Psychiatric Symptoms in Wilson’s Disease-Consequence of *ATP7B* Gene Mutations or Just Coincidence?-Possible Causal Cascades and Molecular Pathways. Int. J. Mol. Sci..

[36] Berk M., Dean O., Cotton S.M., Gama C.S., Kapczinski F., Fernandes B.S., Kohlmann K., Jeavons S., Hewitt K., Allwang C. (2011). The efficacy of N-acetylcysteine as an adjunctive treatment in bipolar depression: An open label trial. J. Affect. Disord..

[37] Fornaro M., McCarthy M.J., De Berardis D., Pasquale D., Martino M., Colicchio S., Cattaneo C.I., Ngelo D., Fornaro P., Tabaton M. (2013). Adjunctive agomelatine therapy in the treatment of acute bipolar II depression: A preliminary open label study. Neuropsychiatr. Dis. Treat..

